# Medication Adherence in Patients With Glaucoma and Determinants of Treatment Adherence: A Cross-Sectional Study

**DOI:** 10.7759/cureus.116254

**Published:** 2026-09-14

**Authors:** Sujata R Charel, Vishva S Sheth, Stuti S Kothari, Jaini Shah, Punit Singh

**Affiliations:** 1 Ophthalmology, Sumandeep Vidyapeeth Deemed to be University, Vadodara, IND

**Keywords:** anti-glaucoma medication, clinician-patient communication, glaucoma, health behavior, intraocular pressure, medication adherence, patients compliance, treatment barriers, visual impairment

## Abstract

Objective: This study aims to assess the level of medication adherence among patients with glaucoma, examine the association between adherence and glaucoma progression, and identify determinants of adherence.

Methods: This hospital-based, observational, cross-sectional study was conducted over 18 months. A total of 77 patients aged >18 years with glaucomatous changes were included. Data collection involved comprehensive ophthalmic examination and administration of the Glaucoma Adherence Study Questionnaire. Statistical analysis was performed using Epi Info version 7 (CDC, Atlanta, GA).

Results: The mean age was 60.48 ± 13.32 years, with 62.34% males. Based on perimetry results, 24.7% had mild, 40.3% had moderate, and 35.0% had severe glaucoma. Medication adherence was reported as full adherence by 55.8% (43/77) of patients. Significant determinants influencing adherence included glaucoma severity (p = 0.002), education level (p < 0.001), income (p < 0.001), clinician-patient communication (p < 0.001), annual follow-up (p = 0.03), motivation (p < 0.001), memory (p < 0.001), difficulty in drop administration (p = 0.001), and side effects (p < 0.001).

Conclusion: Medication adherence among glaucoma patients was suboptimal at 55.8%. Multiple modifiable factors influenced adherence, highlighting the need for targeted interventions, including patient education, simplified regimens, and improved clinician-patient communication, to enhance treatment outcomes.

Clinical significance: Glaucoma is a chronic progressive optic neuropathy and a leading cause of irreversible blindness. So the primary goal of treatment is to lower intraocular pressure (IOP), usually through long-term use of topical medication.

Poor adherence to prescribed therapy can lead to inadequate IOP control, faster progression of optic nerve damage, visual field deterioration, increased risk of irreversible vision loss and blindness, and greater healthcare costs due to additional treatment and follow-up visits.

## Introduction

Glaucoma represents a significant global health concern as one of the leading causes of irreversible blindness [1]. This group of eye conditions primarily damages the optic nerve, often due to elevated intraocular pressure (IOP) [2]. Effective management is crucial to prevent progressive vision loss, with topical hypotensive agents being the first-line treatment aimed at reducing IOP and halting disease progression [3].

Adherence to medication regimens is critical in managing chronic conditions such as glaucoma [4]. Non-adherence to prescribed medications can result in uncontrolled IOP, increasing the risk of optic nerve damage and subsequent vision impairment [5]. Despite the availability of effective treatments, adherence rates among glaucoma patients are frequently suboptimal, posing substantial challenges to disease management [6].

Several factors influence medication adherence, including patient-related factors such as age and cognitive function, medication-related factors like side effects and regimen complexity, and health system-related factors such as access to care and clinician-patient communication. Understanding these factors is essential for developing strategies to improve adherence and optimize treatment outcomes [7,8].

The present study aimed to assess medication adherence in patients with glaucoma and identify the most common factors influencing adherence to develop targeted interventions for improving treatment outcomes.

## Materials and methods

This hospital-based, observational, cross-sectional study was conducted over an 18-month duration. The study received approval from the Institutional Ethics Committee.

Inclusion criteria

Patients aged >18 years with glaucomatous optic disc changes, glaucomatous visual field changes, patients with normal or high IOP, and those taking glaucoma medications were included in the study.

Exclusion criteria

Patients not consistent with follow-up, uncooperative with questionnaires, having difficulty in examination, patients with corneal pathology, previous ocular surgery, or ocular infections, and patients unwilling to participate were excluded from the study.

Sample size calculation

The sample size was calculated using the hypothesis testing method with the formula $N = \frac{Z^{2} \, p (1-p)}{L^{2}}$, where *Z* = 1.96 at a 95% confidence interval, *p* = 78% (the prevalence of adherence was derived from a previous study by Bhat et al. [9]), and *L* = 10% margin of error. The calculated minimum sample size was 69, inflated by 10% for non-response rate, resulting in 77 patients.

Data collection

Data were collected using a pre-designed semi-structured proforma capturing socio-demographic details and ophthalmic measurements. The Glaucoma Adherence Study Questionnaire was used to evaluate medication adherence and identify related determinants [10]. The questionnaire assessed medication use patterns, barriers to adherence, patient beliefs and attitudes, support systems, self-efficacy, and communication with healthcare providers.

Ophthalmic examination

All participants underwent a comprehensive examination, including visual acuity measurement (uncorrected visual acuity and best-corrected visual acuity), IOP measurement using non-contact tonometry, gonioscopy, pachymetry, fundus examination, and perimetry using Humphrey Field Analyzer with 24-2 SITA standard (SS) program.

The duration of glaucoma and glaucoma diagnosis were recorded. The study population included patients with primary open-angle glaucoma, primary angle-closure glaucoma, normal-tension glaucoma, juvenile open-angle glaucoma, and primary angle closure. Glaucoma severity was categorized as mild, moderate, or severe based on visual field assessment. Glaucoma severity was classified according to the Hodapp-Parrish-Anderson visual field criteria. Mild glaucoma was defined by a mean deviation (MD) better than −6 dB, moderate glaucoma by an MD between −6 and −12 dB, and severe glaucoma by an MD worse than −12 dB. Visual field assessment was performed using automated perimetry, and the severity category was assigned based on the visual field findings.

Adherence questionnaire

The Glaucoma Adherence Study Questionnaire assessed patient adherence to glaucoma treatment regimens [10]. This structured tool included questions focusing on various aspects of medication adherence, such as the frequency and timing of medication use, factors influencing adherence, and potential barriers to following prescribed treatment plans.

The questionnaire comprehensively assessed multiple factors related to glaucoma medication adherence. It evaluated medication-use patterns by asking patients about the frequency of medication intake, adherence to prescribed dosage schedules, and any missed doses. Potential barriers to adherence were explored, including forgetfulness, medication side effects, inadequate understanding of the treatment regimen, financial constraints, and difficulties in obtaining medication refills. The questionnaire also examined patients’ beliefs and attitudes toward glaucoma and its treatment, including their understanding of the disease, perception of the importance of medication adherence, and awareness of the potential consequences of poor adherence. In addition, information regarding support systems was collected to determine the extent of assistance provided by family members, friends, or healthcare professionals in facilitating treatment adherence. Patients’ self-efficacy, defined as their confidence in correctly following the prescribed treatment regimen, was also assessed. Furthermore, the questionnaire evaluated the quality and frequency of communication between patients and healthcare providers, including discussions related to treatment plans, disease management, and challenges encountered in maintaining adherence.

Statistical analysis

Data were entered and analyzed using Epi Info version 7 (CDC, Atlanta, GA). Continuous variables were expressed as mean ± standard deviation. Categorical variables were expressed as proportions. The chi-square test was used for categorical variables and the unpaired t-test for continuous variables. A p-value <0.05 was considered statistically significant. As the analysis was exploratory, no adjustment for multiple comparisons was performed.

## Results

The study included 77 glaucoma patients with a mean age of 60.48 ± 13.32 years and male predominance (62.34%). The mean duration of glaucoma was 11.86 ± 6.668 years, with mean IOP values of 17.56 ± 3.44 mmHg for the right eye and 17.95 ± 3.73 mmHg for the left eye (Table 1).

**Table 1:** Demographic and clinical characteristics of study participants (n = 77) (mean ± standard deviation). IOP: intraocular pressure; CDR: cup-to-disc ratio.

Parameter	Value
Age (years)	60.48 ± 13.32
Male gender	48 (62.34%)
Female gender	29 (37.66%)
Duration of glaucoma (years)	11.86 ± 6.668
IOP, right eye (mmHg)	17.56 ± 3.44
IOP, left eye (mmHg)	17.95 ± 3.73
CDR, right eye	0.713 ± 0.1068
CDR, left eye	0.725 ± 0.1053

Primary open-angle glaucoma (POAG) was the most common diagnosis (63.64%), followed by primary angle-closure glaucoma (PACG) at 20.78%. Based on perimetry findings, moderate glaucoma was most prevalent (40.3%), followed by severe (35.0%) and mild (24.7%) cases (Table 2).

**Table 2:** Distribution of glaucoma types and severity (%). POAG: primary open-angle glaucoma; PACG: primary angle-closure glaucoma; NTG: normal-tension glaucoma; JOAG: juvenile open-angle glaucoma; PAC: primary angle closure.

Parameter	Frequency (%)
Diagnosis	
POAG	49 (63.64%)
PACG	16 (20.78%)
NTG	8 (10.39%)
JOAG	3 (3.90%)
PAC	1 (1.30%)
Severity (perimetry)	
Mild	19 (24.7%)
Moderate	31 (40.3%)
Severe	27 (35.0%)

Overall medication adherence was 55.8%, with 44.2% of patients reporting non-adherence in the past two weeks. In the last two days, 33.77% of patients missed no drops, while 66.23% missed at least one drop (Table 3).

**Table 3:** Medication adherence patterns (%).

Parameter	Frequency (%)
Adherence in the past 2 weeks	
Fully adherent	43 (55.8%)
Non-adherent	34 (44.2%)
Drops missed in the last 2 days	
No drops missed	26 (33.77%)
1 drop	23 (29.87%)
2 drops	15 (19.48%)
3 drops	8 (10.39%)
4 drops	5 (6.49%)

Treatment-related barriers significantly affected adherence. Difficulty in drop administration was reported by 53.25% of patients, with significantly higher prevalence among non-adherent patients (88.2% vs. 25.6%, p = 0.001). Side effects were experienced by 46.75% of patients and were strongly associated with non-adherence (Table 4).

**Table 4:** Treatment-related factors affecting adherence (%).

Factor	Frequency (%)	Adherent (n = 43)	Non-adherent (n = 34)
Difficulty administering drops	41 (53.25%)	11 (25.6%)	30 (88.2%)
Unsure about proper administration	23 (29.87%)	1 (2.3%)	22 (64.7%)
Disturbing side effects	36 (46.75%)	8 (18.6%)	28 (82.4%)
Prescribed >1 type of drops	39 (50.65%)	15 (34.9%)	24 (70.6%)

Regarding lifestyle busyness, 28.57% reported being normally busy, 58.44% were not very busy, and 12.99% were very busy. In terms of living arrangements, 12.99% lived alone, 42.86% lived with a partner, 9.09% lived with young children, and 31.17% lived with adult children. When rating their overall health, 22.08% felt it was a little bit better, 37.66% felt it was the same, another 37.66% felt it was a little bit worse, and 15.58% felt it was much worse. For motivation to take drops, ratings were distributed as follows: 10.39% rated 3, 19.48% rated 4, 11.69% rated 5, another 19.48% rated 6, 22.08% rated 7, and 16.88% rated 8. Regarding memory, 6.49% rated 2, another 6.49% rated 3, 14.29% rated 4, 27.27% rated 5, another 14.29% rated 6, 7.79% rated 7, 22.08% rated 8, and 1.30% rated 9. Regarding diagnoses, 1.30% were diagnosed with dementia, while 98.70% were not. No patients were diagnosed with depression.

Patient understanding significantly influenced adherence. Patients with an understanding that treatment prevents vision deterioration exhibited better adherence (51.2% vs. 41.2%), while 50% of non-adherent patients believed treatment would "not do much" compared to only 7% of adherent patients (chi-square = 10.79) (Table 5).

**Table 5:** Patient awareness and beliefs (chi-square = 10.79).

Factor	Adherent (n = 43)	Non-adherent (n = 34)	P-value (chi-square = 10.79)
Understanding treatment goals			
Prevent deterioration of vision	22 (51.2%)	14 (41.2%)	0.002
Improve vision	9 (20.9%)	3 (8.8%)	<0.001
Not do much	3 (7.0%)	17 (50.0%)	<0.001
Believe drops control progression	33 (76.7%)	13 (38.2%)	<0.001
Satisfied with glaucoma education	29 (67.4%)	22 (64.7%)	0.24

Higher education and income levels were significantly associated with better adherence. All patients with no schooling were non-adherent, while 37.2% of adherent patients had higher education. Middle-income patients (1-10 lakhs) showed the best adherence (72.1%), and regular annual follow-up was associated with better adherence (%) (Table 6).

**Table 6:** Socioeconomic and healthcare factors (%).

Factor	Adherent (n = 43)	Non-adherent (n = 34)
Education		
No schooling	0 (0%)	6 (17.6%)
Primary	8 (18.6%)	18 (52.9%)
Secondary	19 (44.2%)	8 (23.5%)
Higher education	16 (37.2%)	2 (5.9%)
Annual income		
<1 lakh	7 (16.3%)	23 (67.6%)
1-10 lakhs	31 (72.1%)	10 (29.4%)
>10 lakhs	5 (11.6%)	1 (2.9%)
Yearly follow-up done	24 (55.8%)	10 (29.4%)

Difficulty in understanding the doctor significantly influenced adherence, as patients who never found it difficult to understand their doctor adhered more compared to those who found it most difficult. Similarly, difficulty in communicating with the doctor showed a significant difference, with patients who never found it difficult to communicate adhering more compared to those who found it most difficult. Yearly follow-up had a significant impact, with patients who had yearly follow-ups adhering more compared to those who did not.

There was no significant difference in adherence related to travel time to the treating center. However, those with a normally busy lifestyle had higher adherence, although the difference was not significant. A significant difference was found in living situations, where patients living with a partner had higher adherence compared to those living alone. Overall health rating showed significant differences, with patients who felt their health was a little bit better adhering more than those who felt their health was much worse.

Motivation to take drops was significantly associated with adherence, with higher motivation ratings correlating with higher adherence. Memory rating also showed a significant difference, with patients having higher memory ratings adhering more. Understanding treatment goals was another significant factor, as patients who believed the goal was to keep their vision from getting worse had higher adherence. Belief in the importance of taking drops significantly influenced adherence, with those believing in the importance adhering more.

There was no significant difference in beliefs about blindness treatment. However, belief that drops could control glaucoma progression was significant, with higher adherence among those who believed in this. Satisfaction with glaucoma education did not show a significant difference. Difficulty in putting drops in properly was a significant factor, with patients who did not find it difficult to put in drops adhering more. Feeling unsure about how to properly use drops also showed a significant difference, with those not feeling unsure adhering more. Finally, the presence of disturbing side effects from drops significantly affected adherence, with those without disturbing side effects adhering more.

## Discussion

The present study found medication adherence of 55.8% among glaucoma patients, which falls within the wide range (24-98%) reported in the literature [11]. The observed suboptimal adherence rate is concerning, given the progressive nature of glaucoma and the pivotal role of sustained medication adherence in preventing vision loss.

In our study, the mean age of 60.48 ± 13.32 years was slightly higher than that reported by Bhat et al. [9] (53.86 ± 8.04 years) and Kim et al. [12] (55.1 years). The male predominance (62.34%) aligns with findings from Rajurkar et al. [13] (62.5%) and Spencer et al. [14] (58%). The mean disease duration was longer at 11.86 years compared to Kim et al. [12].

In our study, treatment-related factors emerged as significant barriers to adherence. Difficulty in drop administration affected 53.25% of patients and was strongly associated with non-adherence (p = 0.001). This contrasts with Rajurkar et al. [13], who found administration difficulty insignificant. The high prevalence of side effects (46.75%) significantly impacting adherence (p < 0.001) highlights the need for strategies to manage adverse effects and alternative formulations.

In our study, patient understanding and beliefs played crucial roles in adherence. Those who correctly understood treatment goals showed significantly better adherence (p < 0.001). The finding that 50% of non-adherent patients believed treatment would "not do much" emphasizes the importance of patient education. This aligns with Birhanie et al. [15], who reported that 85.4% of adherent patients were motivated by fear of blindness.

In our study, socioeconomic factors strongly influenced adherence. Higher education levels were associated with better adherence (p < 0.001), though this contrasts with some studies finding no association. The significant impact of income (p < 0.001) corroborates findings by Bhat et al. [9], suggesting financial stability facilitates medication access.

In our study, the association between regular follow-up and adherence (p = 0.03) emphasizes the importance of continuous monitoring. Better clinician-patient communication was significantly associated with adherence (p < 0.001), supporting the need for clear, empathetic communication in chronic disease management.

Limitations

The cross-sectional design prevents establishing causality. The single-center setting may limit generalizability. Patients with inconsistent follow-up were excluded from the study. This may have introduced selection bias because patients with poorer medication adherence may be more likely to miss scheduled follow-up visits. The study evaluated multiple potential factors associated with medication adherence using univariate analyses without adjustment for multiple comparisons or multivariable regression. Therefore, some statistically significant findings may represent chance associations, and the observed associations should not be interpreted as independent predictors.

## Conclusions

Medication adherence among glaucoma patients was suboptimal at 55.8%, with multiple modifiable factors influencing adherence. Treatment-related challenges, patient awareness, socioeconomic status, and healthcare factors significantly affected adherence. Targeted interventions addressing these barriers through patient education, simplified regimens, side effect management, and improved clinician-patient communication could enhance adherence and treatment outcomes. Future prospective studies with objective adherence measures and multiple visual field assessments are needed to establish the relationship between adherence patterns and disease progression.
